# Hereditary Hyperferritinemia-Cataract Syndrome: A Pediatric Case Without Congenital Cataract

**DOI:** 10.7759/cureus.95062

**Published:** 2025-10-21

**Authors:** Anusha Hemanna, Richard Sidlow

**Affiliations:** 1 Medicine, Mandya Institute Of Medical Sciences, Mandya, IND; 2 Medical Genetics and Metabolism, Valley Children's Hospital, Madera, USA

**Keywords:** congenital cataract, hereditary hyperferritinemia, iron overload, iron responsive element, light ferritin gene

## Abstract

The differential diagnosis for hyperferritinemia is wide, including malignancy, infections, autoimmune disorders, hemophagocytic lymphohistiocytosis, hyperthyroidism, chronic kidney disease, and, most commonly, iron overload. As an acute-phase reactant, ferritin is usually elevated due to secondary causes. In rare circumstances, however, ferritin levels may be primarily elevated due to a genetic cause. We report a case of a three-year-old male patient with incidentally detected hyperferritinemia who was found to harbor the c.-168G>T mutation in the FTL gene, confirming hereditary hyperferritinemia-cataract syndrome (HHCS). This case is distinguished by a unique four-generation family history of early-onset cataracts and elevated ferritin levels, underscoring the hereditary and novel nature of this disorder.

## Introduction

The uncommon condition known as hereditary hyperferritinemia-cataract syndrome (HHCS) is typified by elevated serum ferritin levels, congenital bilateral cataracts, and no tissue iron overload [1,2]. Otherwise known as Bonneau-Beaumont Syndrome, this syndrome was initially identified as an autosomal dominant hereditary disease in 1995 by two separate research teams in France and Italy [3]. An important feature of HHCS is the progressive cataracts with highly distinctive morphology [4]. Approximately one in 200,000 people has the condition. However, this figure is likely understated since, even in cases where iron overload is not present, the illness is sometimes misdiagnosed as hereditary hemochromatosis because of hyperferritinemia.

The iron-responsive element (IRE) found upstream of the light ferritin gene (FTL) is mutated in HHCS. The gene product of a classical IRE is an mRNA structure that post-transcriptionally controls the production of proteins involved in iron metabolism by interacting with the iron regulatory proteins (IRP1 and IRP2). The FTL gene itself has been shown to contain at least 47 mutations that cause HHCS, including 36 single-nucleotide mutations, nine deletions, and two insertion-deletions [5]. Families with HHCS have been found to have at least 25 distinct genetic changes, including those affecting the complete IRE structure. There have also been reports of isolated instances brought on by de novo mutations [6]. While some of these genetic changes impact the stems or the bulge of the IRE structure and change its conformation, others disrupt the loop that directly interacts with iron regulatory proteins, which result in the uncontrolled synthesis of ferritin and inhibit iron binding [7,8].

While cataracts are the cardinal and well-documented ocular manifestation of HHCS, the correlation between genotype and the variability of the ocular phenotype, including factors such as age of onset and severity of cataracts, remains poorly understood [4]. Broader awareness and early diagnosis of this entity when hyperferritinemia is detected have the potential to improve visual outcomes for patients.

## Case presentation

Our patient first presented to his general pediatrician at 18 months of age due to parental concern for hyperpigmentation outside of the context of increased sun exposure. Prior complaints included pale skin, frequent bruising, and occasional epistaxis and intermittent fluctuations in activity levels, but denied any blood transfusions, chronic illnesses, surgeries, or hospitalizations. Pregnancy, birth, and developmental histories were all unremarkable.

As part of the workup for his hyperpigmentation, a complete blood count and iron studies were conducted. Mild iron-deficiency anemia was detected, and the patient was started on oral ferrous sulfate. Treatment was discontinued after a month as his skin color returned to normal and his blood counts normalized. Incidentally, serum ferritin levels were found to be elevated, ranging from 1916.9 ng/ml (normal 7-140ng/ml), and the reactive thrombocytosis was secondary to iron deficiency during the time of investigation. The laboratory reports carried out have been listed in Table 1.

**Table 1:** Laboratory reports MCV: mean corpuscular volume; WBC: white blood cell

Component	Value	Reference value
Hemoglobin (g/dl)	13.3	13-18
MCV (fl)	79.7	74-87
Platelet (10^9^/L)	537	150-450
Absolute neutrophils (cells/microliter)	6,109	2,500-7,000
Serum ferritin (ng/ml)	1916.9	7-140
WBC (10^9^/L)	12.9	5-19

**Figure 1:**
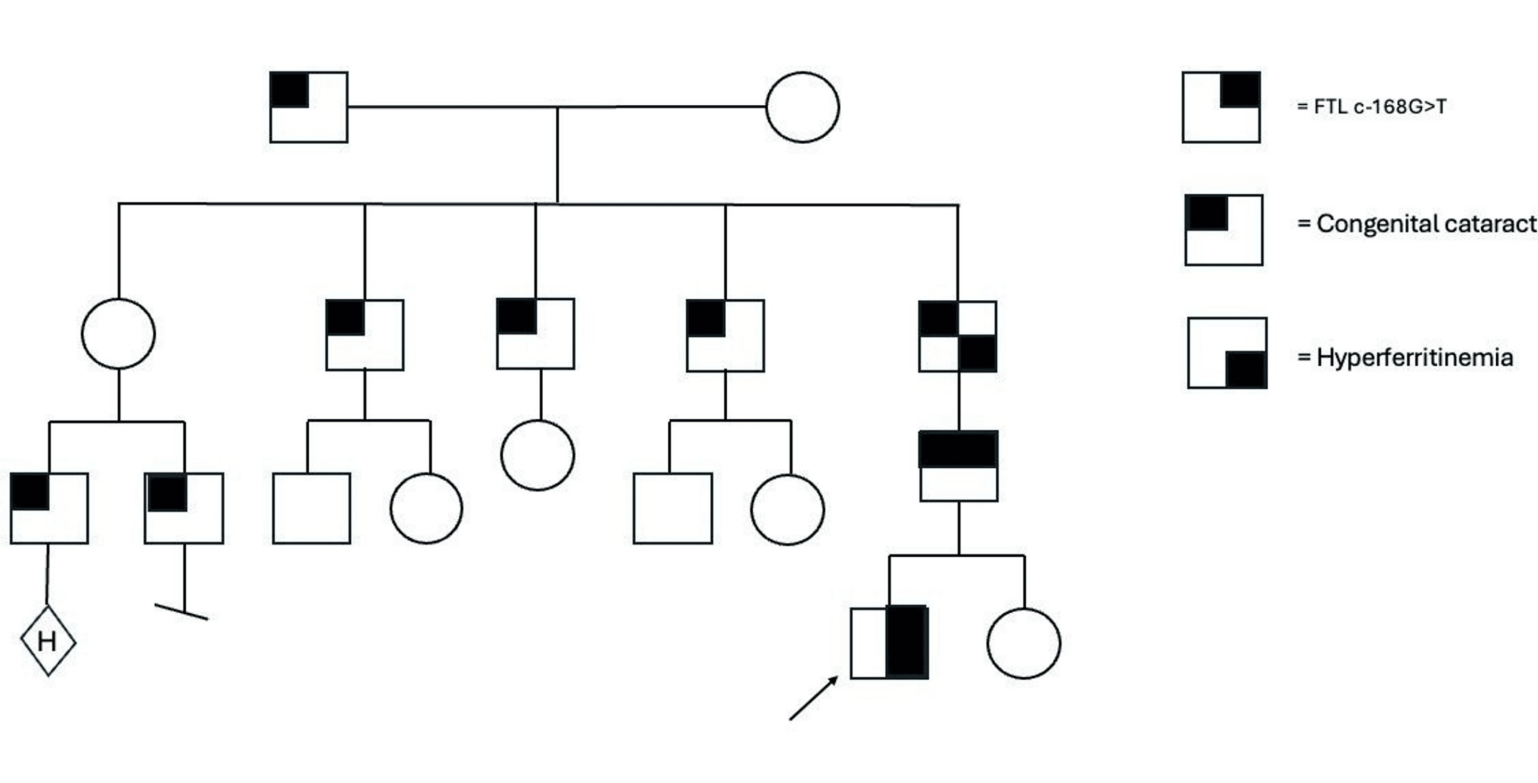
Pedigree analysis of hereditary hyperferritinemia-cataract syndrome The family pedigree demonstrates a pattern of inheritance consistent with autosomal dominance, with affected male members observed in four successive generations. The arrow indicates the individual evaluated in the current study

A hereditary hemochromatosis panel (Invitae) was performed, which revealed our patient to be a heterozygous carrier for the H63D variant of the HFE gene. At age three, our patient's genetic consultation was obtained. Vital signs, growth parameters, and physical examination were within normal limits except for a bruise on his upper left arm. Family history was positive for father, paternal grandfather, paternal paternal great-grandfather, three paternal great uncles, and two paternal first cousins once removed with congenital cataracts, paternal grandfather with hyperferritinemia (Figure 1-pedigree). No history of hematologic malignancies, myelodysplasias, hemochromatosis, bleeding disorders, or clotting disorders was documented in the family.

Whole-genome sequencing, including intronic analysis of the ferritin light chain (FTL) gene (Variantyx), revealed a heterozygous variant, c.-168G>T (rs398124635), in the proband, with cascade testing revealing the same variant in the proband’s father but not in his mother or sister. The c.-168G>T variant in the FTL gene is pathogenic for autosomal dominant hyperferritinemia-cataract syndrome based on American College of Medical Genetics and Genomics (ACMG) criteria. It is supported by strong evidence of disruption in a critical gene region (PP1), previous reports linking it to the syndrome (PS3), and co-segregation with disease in affected family members consistent with autosomal dominant inheritance (PM2). These ACMG-based findings highlight the importance of genetic testing in diagnosing this rare disorder. 

## Discussion

The first documented genetic disease caused by regulatory alterations influencing translation was hereditary HHCS. It so constitutes a new mechanism of cataract development. HHCS leads to cataract formation due to the accumulation of abnormal L-ferritin protein aggregates in the lens, caused by mutations in the FTL gene's IRE regulatory region. Several publications have documented mutations that impact the FTL IRE and result in HHCS since its discovery in 1995. Initial presentations of HHCS vary based on age, causal variant in FTL, and the presence of homozygosity/combined heterozygosity of C282Y and/or H63D variants in the HFE gene. Investigations have also demonstrated that HHCS is caused by specific chromosomal mutations in the IRE of the L-ferritin subunit, which result in the uncontrolled synthesis of ferritin and inhibit iron binding, which produces negative feedback (Table 2) [8].

**Table 2:** Case review of HHCS HHCS: hereditary hyperferritinemia-cataract syndrome; FTL: ferritin light chain; IRE: iron-responsive element

Authors	Demographic details	Clinical presentation	Family history	Lab investigations	Genetic diagnosis
Neofytou et al. (2024) [9]	13-year-old girl	Normal medical history and clinical examination. Ophthalmological assessment revealed mild lens opacity	Nil	Persistent hyperferritinemia (1437 ng/ml). Transferrin saturation- 28.5%. Normal serum ferritin: 7-142 ng/mL	c.-161C>G mutation in the FTL gene (heterozygous)
Alvarenga et al. (2022) [10]	45-year-old woman	Bilateral cataracts diagnosed at 25, worsening glare, and cataract surgery in her fifties	72-year-old mother had SF: 1,703 ng/mL and 2,145 ng/mL with normal iron parameters and no hepatic iron overload. 11-year-old son had SF: 1,260 ng/mL and 1,308 ng/mL	Hyperferritinemia (1,487 ng/mL), iron levels (100 mcg/dL), transferrin saturation-28%. Liver MRI showed no iron overload (1.1 mg/g). Normal serum ferritin: 24-307 ng/mL	HFE gene mutations (p.Cys282Tyr, p.His63Asp) were absent c.-164C>G in positive 5’UTR region
Celma Nos et al. (2021) [2]	Case 1: 38-year-old woman	Unexplained hyperferritinemia	Maternal relatives with hyperferritinemia and cataracts, along with early ischemic deaths	Hyperferritinemia (1143 ng/mL). Normal serum ferritin: 24-307 ng/mL	Heterozygous c.-160A>G mutation in the FTL gene’s IRE region, altering the conserved apical hexanucleotide loop (CAGUGU)
	Case 2: 44-year-old woman	Vision issues, leading to a cataract diagnosis and surgery at 44	Mother, aunt, brother, and niece also had hyperferritinemia and cataracts, with some undergoing cataract surgery	Hyperferritinemia (919 ng/mL) Normal serum ferritin: 24-307 ng/mL	Heterozygous for NM_00146.3: c.[-167C>T]. A mutation in the FTL gene also known as +33c>T Madrid / Philadelphia pathological variant. Mutation is located in c-bulge of FTL IRE
Christiansen et al. (2007) [8]	9-year-old boy	Blurred vision for three months, affecting his daily activities. Slit-lamp examination revealed bilateral cataracts with central cortical and nuclear opacities in a stellate pattern	Autosomal-dominant juvenile cataracts, as his mother and two maternal aunts had early-onset cataracts with high serum ferritin	Hyperferritine mia (1013 µg/L). Normal serum ferritin: 7-142 ng/mL	Clinical diagnosis, not genetically confirmed
	10-year-old girl	Progressive vision loss was referred for evaluation of recently diagnosed bilateral cataracts. Her uncorrected visual acuity was 20/40 in both eyes	Multiple generations with early-onset cataracts.	Normal iron levels and binding capacity, but elevated ferritin 555 µg/L. Normal serum ferritin: 7-142 ng/mL	Clinical diagnosis, not genetically confirmed
Morais et al. (2023) [11]	4-year-old boy	Ophthalmologic evaluation revealed bilateral anterior and posterior subcapsular lens vacuoles. His visual acuity was 20/20 in the right eye and 20/40 in the left, with a normal fundus	First-degree relatives showed normal ferritin levels	Hyperferritinemia (1056 ng/mL) Normal serum ferritin: 7-142 ng/mL	Clinical diagnosis, not genetically confirmed
Serra et al. (2011) [12]	3-year-old boy	Poor appetite, laboratory tests revealed unexplained hyperferritinemia. At diagnosis, the child had no visual deficits or lenticular opacities but remains under regular follow-up for early cataract detection	Father had H63D/C282Y heterozygosity and confirmed genetic hemochromatosis. Family history revealed high ferritin levels and bilateral congenital cataracts in multiple maternal relatives, requiring surgery in some	Hyperferritinemia (600 μg/L). Subsequent tests confirmed increasing ferritin levels (up to 1247μg/L). Normal serum ferritin: 7-142 ng/mL	Heterozygous H36D in HFE A37C mutation in the L-ferritin gene

Our patient was diagnosed by chance during the anemia workup with HHCS after laboratory testing revealed hyperferritinemia with concurrent iron overload (high ferritin), the causes of secondary iron overload (alcohol use disorder, nonalcoholic fatty liver disease, and hepatitis C virus) were ruled out, and a positive family history of HH (our genetic analysis revealed a heterozygous carrier status for the H63D variant of the HFE gene) and cataract was elicited. Our case involves four generations of exclusively male individuals, many of whom presented with congenital cataracts. Despite the segregation pattern looking X-linked in nature, the FTL gene lies on chromosome 19 and is inherited in an autosomal fashion. 

Unlike Neofytou et al. (Table 2), who reported a sporadic case, our study identified a significant family history spanning four generations with congenital cataracts predominantly affecting males [9]. Furthermore, our genetic analysis revealed a heterozygous carrier status for the H63D variant of the HFE gene, contrasting with Alvarenga et al. (Table 2), who ruled out HFE gene mutations [10]. Notably, our study also found hyperferritinemia in first-degree relatives, differing from Morais et al. (Table 2), who reported normal ferritin levels in family members [11]. These differences highlight the complexity and variability of this condition. Serra et al. reported on a pediatric case of HHCS that did not present with cataracts; this case, unlike our case, involved exclusively maternal relatives of the male proband harboring a different variant (A37C) in FTL (Table 2) [12]. Our case involves four generations of exclusively male individuals, many of whom presented with congenital cataracts. Most other cases in the medical literature do not display such extreme gender specific segregation, which argues against an imprinting effect or segregation patterns being affected by the presence of specific exonic versus intronic variants driving the phenotype. However, given the paucity of cases reported to date, the preceding cannot be definitively ruled out. Additionally, there does not seem to be a correlation between the presence of a specific exonic versus intronic variant to explain the presence of congenital versus later onset of cataracts. However, genotype/phenotype correlation may be achievable based on measurement of time course of elevated ferritin levels as they relate to the specific variants known to cause HHCS to see if absolute levels or initiation/length of exposure to high ferritin levels may explain such this difference in phenotype (cataract formation in this syndrome being due to 10x elevation of ferritin levels within the lens).

Since hyperferritinemia is a biochemical indicator shared by both hereditary hemochromatosis and HHCS, the two conditions are misdiagnosed. This would require a high level of suspicion for this ultrarare disease amongst general practitioners, ophthalmologists, and gastroenterologists, with subsequent referral to geneticists to direct the genetic workup appropriately and avoid unnecessary phlebotomy for diagnosis or treatment (Table 2) [13].

Further research into sequence variants and/or alterations in methylation or other epigenetic signals in other components of the iron regulation system could explain the variable timing and clinical presentations of this syndrome and shed further light on this complex process.

## Conclusions

Congenital, pediatric or early-onset cataracts, or unexplained hyperferritinemia in the absence of iron overload, either in isolation or in combination in an individual or a family, should raise clinical suspicion of HHCS. The diagnosis of HHCS was made after three generations of the patient's family showed an autosomal dominant inheritance pattern of hyperferritinemia with a history of cataracts. Hence, early identification of uncommon hereditary disorders is made possible by family history, which is a crucial screening tool for interpreting abnormal clinical findings. Our case, with its four-generation transmission primarily affecting males, raises the possibility of additional biological mechanisms, such as imprinting effects or sex-linked genetic/environmental modifiers, which merit further study. This case not only supports the importance of early diagnosis of HHCS but also suggests the need for further studies to elucidate the mechanisms of phenotypic variability, such as the age of cataract onset and the potential role of modifying factors. Further elucidation of these factors may lead to improvements in patient care and guide targeted therapy in the future. Prompt referral to a geneticist for directed testing of both the exonic and 5’-intronic space covering the FTL gene should follow the detection of these clinical findings and/or family history. Further research into the role of known variants causing this disease and clarification of genotype/phenotype correlations could reveal further insights into the iron regulation system, which is warranted.
